# Evaluation of the Relationship between Oral Hygiene Index Simplified (OHI-S) and Oral Health Behavior: Relationship between Oral Hygiene Index Simplified and Oral Health Behavior

**DOI:** 10.31661/gmj.v13iSP1.3639

**Published:** 2024-12-08

**Authors:** Zeynab Moghaddami, Mojgan Faezi, Hassan Semiyari

**Affiliations:** ^1^ Guilan University of Medical Sciences, Guilan, Iran; ^2^ Department of Community Oral Health, School of Dentistry Shahed University, Tehran, Iran; ^3^ Department of Periodontics, School of Dentistry Shahed University, Tehran, Iran

**Keywords:** OHI-S Index, Oral Health-related Behavior (OHB), Dentists

## Abstract

Researchers aimed to explore oral health habits among medical students from diverse backgrounds. The primary focus was to understand the connection between the OHI-S index and oral health behaviors in Shahed University students in 2022.In this descriptive study, 175 participants from nursing, operating room, and dental programs were examined using the OHI-S index. Data collection involved demographic and oral health behavior questionnaires. Through simple random sampling, and after obtaining written consent, oral examinations were conducted. Researchers carefully assessed and recorded participants’ oral health status using the OHI-S criteria. The findings revealed interesting results: Dental students excelled in the plaque index, followed by nursing and operating room students. In terms of the calculus index, nursing students led, followed by dental and operating room students. Statistical analysis indicated a significant association between the field of study and the OHI-S index. Dental students achieved higher scores compared to their peers. Additionally, there was a notable relationship between gender and oral health behaviors, with females demonstrating better practices than males. In conclusion, the study highlights the impact of education on improving oral health habits. Dental students’ specialized knowledge contributed to their superior oral hygiene routines. These findings can be valuable in promoting oral health awareness among various medical student groups.

## Introduction

Today, oral health holds as much importance as general bodily health [1]. Cultural, economic, and social factors significantly influence oral hygiene, which is a personal matter [2]. The World Health Organization (WHO) asserts that oral hygiene is an essential component of physical and mental well-being, shaped by the values and attitudes of individuals and societies [3].

Studies indicate that tooth decay prevalence among students in certain areas surpasses World Health Organization standards. Student life comes with unique circumstances, including being away from family, living in an unfamiliar city, limited access to adequate health amenities, exposure to diverse cultures, and high living costs. These factors, along with irregular sleep patterns, impact students’ oral health behaviors [4]. Within the medical sciences, dental students stand out due to the future importance of their profession for community health. They play a crucial role in enhancing oral health. Consequently, dental students are expected to set an example with their behavior, potentially foreshadowing their future role in promoting oral health in society [5]

The OHI-S index offers a swift method to assess oral health [6]. It serves as a valuable tool in epidemiology and dental evaluation programs, aiding professionals in effectively measuring oral health within communities [7]. Developed by Greene and Vermillion in 1960, the OHI classified and evaluated oral health. While the OHI was simple, sensitive, and useful, it was also time-consuming, leading to efforts to create a simpler version with equal sensitivity, the OHI-S. This clinical evaluation allowed for a more precise assessment of students’ oral health behaviors [8].

## Materials and Methods

This cross-sectional study evaluated the oral and dental hygiene practices of medical students using a valid questionnaire. The study group included 175 nursing, operating room and dental students at Shahed University in 2022. After obtaining the code of ethics (IR.Shahed. Rec.1398.044) and necessary permits, data collection began with a reliable questionnaire, which was shown by Cronbach’s alpha of 0.81.

Participants completed a questionnaire that included demographic information and oral hygiene measures. In this study, oral health behavior questionnaire, approved by Khami et al., was used.

The students completed the questionnaire and then the OHI-S assessment was done

To standardize clinical examination conditions, disposable dental instruments such as probes, mirrors and periodontal probes were used on a regular chair with natural daylight from 9:00 AM to 12:00 PM. Following the principles of OHI-S evaluation, one surface of 6 teeth (buccal surfaces of incisors and special molars) was examined. Scoring was as follows: 0 for no plaque or calculus, 1 for plaque or calculus on 1/3 of the tooth surface, 2 for 2/3, and 3 for the entire surface. These scores were summed and divided by the number of levels

The OHI-S index was calculated by averaging and dividing the account and license plate indices. Scores were classified as good, average, or poor. The oral health questionnaire consisted of 15 questions that were classified on a 5-point scale with a total score from 0 to 75 as poor, average, or good performance.

Data analysis was done using SPSS software version 29 and Chi-square and Mann-Whitney tests for statistical inference.

## Results

**Table:** Table1. Pattern of Oral Hygiene Status of Students

**Poor**		**Moderate**		**Good**		**OHI-S**
**Frequency**	**Number**	**Frequency**	**Number**	**Frequency**	**Number**	**Field of Study**
33/33	1	31/08	23	55/10	54	**dentistry**
33/33	1	33/78	25	10/20	10	**Operating room**
33/33	1	35/14	26	34/70	34	**nursing**
100	3	100	74	100	98	**total**

**Table:** Table2. Students Attitude Toward Oral Health Care

					**Answer**					
Questions	**Completely agree agree**		**Agree**		**Disagree**		**Completely agree disagree **		**I don’t know**	
	**frequency**	**percentage**	**frequency**	**percentage**	**frequency**	**percentage**	**frequency**	**percentage**	**frequency**	**percentage**
Adding fluoride to drinking water is an effective way to prevent decay	58	33/14	80	45/71	12	6/85	10	5/72	15	8/58
The number of times the sugar is consumed in causing caries is more than the total amount consumed	47	26/85	78	44/58	23	13/14	19	10/85	8	4/58
Figure sealant therapy is an important factor in preventing peat and fissure of newly erupted teeth	15	8/58	21	12	33	18/85	36	20/57	70	40
The probability of losing a tooth that has undergone restorative treatment is greater than the number of teeth extracted	100	57/14	65	37/14	2	1/14	5	2/86	3	1/72
The use of small amounts of water to wash the teeth after brushing with fluoride causes more impact	20	11/42	20	11/42	97	55/44	25	14/28	13	7/44
Examination of freshly grown tooth grooves with catheters causes enamel damage and more talent for decay	76	43/42	50	28/57	22	12/57	15	8/58	12	6/86
White or brown enamel decay can be seen on the surface of moist tooth enamel has come full-width	57	32/58	41	23/42	27	15/42	35	20	15	8/58
Using fluoride toothpaste is more important than brushing	15	8/58	20	11/42	73	41/71	50	28/58	17	9/71
Oral diseases can lead to systemic diseases	71	40/57	50	28/57	33	18/86	14	8	7	4

Among the 175 participants, 78 (50%) were dental students, 61 (30%) were nursing students, and 36 (20%) were operating room students. The gender distribution was 105 females (60%) and 70 males (40%). Out of the 75 good calculus indicators, 28 were dental, 10 were operating room, and 37 were nursing students. Among the 100 moderate calculus indicators, 50 were dental, 24 were nursing, and 26 were operating room students. No students had poor calculus indicators.

Due to the nature of the variables, the Chi-square test was employed, revealing no significant relationship between the calculus indicator and field of study (P>0.026). The majority of participants (98, 56%) had a moderate OHI-S status. 74 participants (42.28%) exhibited good OHI-S, and 3 (1.71%) had poor OHI-S. Table-1 presents the frequency and percentage of OHI-S by the field of study, demonstrating a significant relationship (P<0.026). The field of dentistry with the highest number of good OHI-S compared to the total population was at the top, followed by nursing and operating room expert, respectively. In Table-2 and Table-3 , there are the frequency and percentage of responses to the oral health behavior questionnaire.

## Discussion

**Table:** Table3. Students behavioural characteristics Toward Oral Health Care

					**Answer**					
**Questions**		**Irregular or never**	**Once a week**	**Two or three times a week **	**Once a day**	**More than once a day**
	**frequency**	**percentage**	**frequency**	**percentage**	**frequency**	**percentage**	**frequency**	**percentage**	**frequency**	**percentage**
You usually brush your teeth every once in a while	91	52	58	33/14	12	6/86	12	6/86	2	1/14
	**Always or almost always **		**Most of the time**		**Rarely**		**Never**	
Do you use fluoride toothpaste when brushing	53	30/28	47	26/88	48	27/42		27		15/42
	**Irregular or never**		**Once a week**		**Two or three times a week **		**Once a day**		**More than once a day**	
How often do you floss once	74	42/28	41	23/42	35	20	15	8/58	10	5/72
	**About three times a day or more **		**About twice a day**		**About once a day**		**Not every day**		**Not every day**	
How often do you eat sugary snacks or sugary drinks between meals	19	10/86	51	29/14	58	33/14	17	9/72	30	17/14
	**Go to the dentist**		**I ask my classmates to do this **		**I do it myself**		**I do not see the need to do this **	
What do you usually do to check your mouth and teeth	49	28	59	33/72	35	20	32		18/28	
	**6 months ago**		**Between 6 months and 12 months **		**During 1 to 2 years**		**2 to 3 years ago5**		**I have not been examined yet **
When was the last check-up of your mouth and teeth	45	25/71	51	29/14	29	16/57	19	10/86	31	17.72

Oral health education and social factors significantly influence oral care. Regarding the training dental students receive, the first factor is crucial, but the social factor also plays a role among other medical groups.This study aimed to compare medical groups regarding oral health behavior and OHI-S. The dental group achieved the highest average score in the questionnaire compared to other disciplines. Additionally, the dental group exhibited better OHI-S status, indicating a significant relationship between OHI-S and oral health behavior, which highlights the impact of education on improving dental students’ attitudes. This, in turn, enhances their oral hygiene practices.

Statistical analysis revealed that dental students’ average scores on the oral health behavior questionnaire were significantly higher than other groups, demonstrating the effect of dental education on improving their attitudes and, consequently, their oral health behavior.

Comparing questionnaire responses with OHI-S showed that students with better OHI-S scores tend to have more regular dental check-ups, brushing, and flossing habits. Dental students consume fewer sugary snacks and drinks, and most use fluoride toothpaste when brushing [2][10].

Considering that children often visit doctors before dentists and that accessing dentists is challenging in some areas, other medical groups should also receive oral health training to provide necessary care to their patients. However, their training protocols currently lack such courses. Therefore, the performance of these students is solely influenced by their family’s culture and socioeconomic level, which is insufficient. It is suggested that medical groups should include oral health education in their curriculum. Nursing students had a better differential index status compared to the dental and operating room groups, and the plaque index was better in the dental group, followed by the nursing and operating room groups, but there was no significant difference between nursing and operating room students.

The average score for male students was lower than that of females, emphasizing the importance of women’s health habits and their increased attention to personal care.

In this study, dental students’ average questionnaire score was lower than that of Indian students, possibly due to differences in the educational system and culture between the two countries, which influence people’s attitudes. However, the average score of the dental students in this study was higher than that of students in Kuwait [12] and Sudan [13], likely due to variations in the educational systems and cultures of these countries. Another reason for this difference is that in Kuwait, students receive training in preventive dentistry and periodontology courses for seven years.

A similar study in Safar, Iran, found a significant relationship between male gender and poor oral hygiene behavior, and between female gender and studying dentistry with a positive attitude. The probability of regular flossing was higher among women than men. With a better attitude, similar to our study, there was a significant relationship between a positive attitude and regular oral hygiene check-ups [14]. Additionally, in Al-Sheikh’s research, the association with female gender was also significant. Consequently, annual evaluations of dental students are crucial for monitoring students and the educational system [15].

## Conclusion

This study highlights that education plays a more significant role in improving oral health behavior compared to social and economic factors. Dental students demonstrated superior oral hygiene practices relative to other groups. Additionally, female students exhibited better oral hygiene behavior than their male counterparts. These findings emphasize the impact of education in health-related fields, as dental students’ specialized training resulted in enhanced attitudes and behaviors towards oral health. This underscores the importance of education within health-related disciplines.

Furthermore, the study reveals that gender can also influence oral health behavior. Women tend to pay closer attention to their health habits, leading to improved oral hygiene practices. These findings can be considered when designing educational and health programs to more effectively influence individuals’ health

## Conflict of Interest

The authors declare no conflict of Intrest with respect to the research, authorship and / or publication of this article.
